# SARS-CoV-2 Infection in a Hippopotamus, Hanoi, Vietnam

**DOI:** 10.3201/eid2903.220915

**Published:** 2023-03

**Authors:** Vuong Nghia Bui, Tung Duy Dao, Long Hoang Tran, Thanh Thi Vu, Trang Huyen Nguyen, Giang Hoang Nguyen, Kien Viet Dung Tran, Huyen Xuan Nguyen, Anh Ngoc Bui, Fred Unger, Hung Nguyen-Viet, Hu Suk Lee

**Affiliations:** National Institute of Veterinary Research, Hanoi, Vietnam (V.N. Bui, T.D. Dao, L.H. Tran, T.T. Vu, T.H. Nguyen, G.H. Nguyen, K.V.D. Tran, A.N. Bui);; International Livestock Research Institute, Hanoi (F. Unger, H.S. Lee);; International Livestock Research Institute, Nairobi, Kenya (H. Nguyen-Viet);; Chungnam National University College of Veterinary Medicine, Daejeon, South Korea (H.S. Lee)

**Keywords:** COVID-19, SARS-CoV-2, severe acute respiratory syndrome coronavirus 2, viruses, respiratory infections, zoonoses, hippopotamus, Vietnam, *Suggested citation for this article*: Bui VN, Dao TD, Tran LH, Vu TT, Nguyen TH, Nguyen GH, et al. SARS-CoV-2 infection in a hippopotamus, Hanoi, Vietnam. Emerg Infect Dis. 2023 Mar [*date cited*]. https://doi.org/10.3201/eid2903.220915

## Abstract

While investigating the death of a hippopotamus at a zoo in Hanoi, Vietnam, we isolated SARS-CoV-2 and sequenced the RNA-dependent RNA polymerase gene from different organs. Phylogenetic analysis showed that the SARS-CoV-2 strain was closely related to 3 human SARS-CoV-2 strains in Vietnam.

On December 4, 2021, a 20-year-old female hippopotamus (*Hippopotamus amphibius*) at a zoo in Hanoi, Vietnam, was treated for lethargy, depression, and reduced appetite. Veterinary staff initiated antimicrobial drug treatment on the basis of the clinical signs. Six days after onset of clinical signs, the hippopotamus was anorexic; she died 17 days after onset. Zoo staff conducted necropsy; the main finding was severe pneumonia. Tissue samples from the liver, spleen, lung, intestine, and blood were collected and sent to the National Institute of Veterinary Research in Hanoi for further diagnosis of viral and bacterial diseases.

We screened the samples by real-time PCR to detect SARS-CoV-2, in accordance with World Health Organization (WHO) PCR protocol (*1*). The lung, spleen, liver, and intestine samples tested positive; cycle threshold (Ct) values for tissue types were 26.67 for lung, 33.53 for spleen, 31.8 for liver, and 36.96 for intestine. No other viral testing was pursued, and tissues were not examined histologically (data not shown).

To obtain the viral isolate, we inoculated the samples into Vero cells according to a method described previously (*2*). After 3 days, we successfully recovered the virus from the lung, spleen, and liver samples (Table). We confirmed that the recovered viruses from Vero cells were SARS-CoV-2 by real-time PCR. We gave the virus the temporary designation SARS-CoV-2/hippo/zoo/Vietnam/2021.

**Table:** Identification and isolation of SARS-CoV-2 from tisue samples of a hippopotamus, Vietnam

Tissue	Real-time RT-PCR result			Virus isolation	
	All betacoronaviruses	SARS-CoV-2		Vero cells	Real-time RT-PCR result
Lung	27.09	26.67		Positive	26.30
Spleen	33.96	33.53		Positive	33.91
Liver	32.29	31.8		Positive	38.34
Intestine	37.84	36.96		Negative	NA
Blood	Negative	Negative		NA	NA

*NA, not applicable; RT-PCR, reverse transcription PCR.

To further characterize and compare the virus isolated from the hippopotamus and the recent human SARS-CoV-2, we used a seminested reverse transcription PCR assay (*3*) to amplify 599–602 bp of the conserved RNA-dependent RNA polymerase (RdRp) genome sequence of 3 human SARS-CoV-2 strains from COVID-19 patients in Vietnam (selected at the same time as the hippopotamus isolate and afterwards) and the isolates from the dead hippopotamus. We sent the purified PCR products to 1^st^ BASE Company (http://www.base-asia.com), Singapore to sequence the 599–602-bp nucleotide of the RdRp genome. We submitted the sequences to GenBank (hippopotamus, accession no. ON365747; human, ON365835–7. We conducted multiple alignments of the obtained sequences of the dead hippopotamus and 3 human COVID-19 patients, together with representative nucleotide sequences of SARS-CoV-2 and other betacoronaviruses available in GenBank, using ClustalW in BioEdit version 7.2.5 as previously described (*4*). We performed phylogenetic analysis in MEGA-X software using the maximum-likelihood method with the best-fit model general time reversible plus gamma 4 plus invariate sites and 1,000 bootstrap replicates (*5*). We constructed a Bayesian maximum-clade credibility host discrete traits tree by using BEAST version 1.10.4 (http://tree.bio.ed.ac.uk/software/beast).

Phylogenetic analysis indicated that the sequences obtained from the dead hippopotamus and 3 human COVID-19 patients were SARS-CoV-2 (Figure; Appendix). The source of the hippopotamus’ infection was difficult to pinpoint because the zoo had been open to the public; a visitor or staff member could have been transmitted the virus. As a precaution, all zoo staff were required to wear uniforms, facemasks, and gloves and to disinfect their boots when servicing the animal areas. However, those biosecurity measures were not sufficient to prevent the airborne transmission of the virus from humans to animals. To prevent anthroponotic disease, zoos must closely monitor the health status of zoo staff to eliminate virus transmission from humans to animals. Active surveillance using nasal or oral swab specimens, or fecal samples from animals, is needed for early detection of viral infection. In addition, stricter biosecurity measures are required in zoo exhibit areas to reduce the potential transmission of viruses by visitors to animals. For example, zoos should install glass barriers to separate exhibit areas from pathways for visitors.

**Figure:**
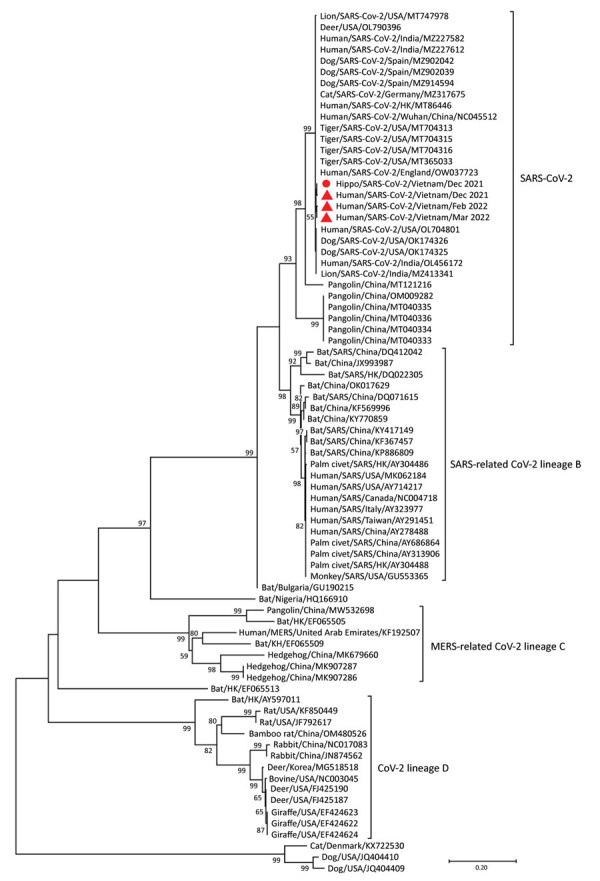
Maximum-likelihood tree constructed for 600-bp RNA-dependent RNA polymerase gene SARS-CoV-2 nucleotide sequences from a hippopotamus (red circle) and 3 human SARS-CoV-2 strains from COVID-19 patients in Vietnam (red triangles) compared with reference betacoronavirus strains obtained from GenBank. Betacoronavirus lineages are indicated on the right of the figure. Scale bar denotes evolutionary distance. MERS, Middle Eastern respiratory syndrome.

This study highlights an urgent need to establish comprehensive monitoring systems for SARS-CoV-2 in animals. Our findings underscore hippopotamuses’ susceptibility to SARS-CoV-2 and further contribute to the knowledge of the epidemiology of SARS-CoV-2, especially regarding the virus’s host range. Whole-genome sequencing will provide information about SARS-CoV-2 lineage to help track transmission pathways. 

Appendix Additional information about SARS-CoV-2 infection in a hippopotamus, Vietnam.
