# Supplementary material for: SARS-CoV-2 Infection in a Hippopotamus, Hanoi, Vietnam
Source: Emerg Infect Dis. 2023 Mar;29(3):658–61. doi: 10.3201/eid2903.220915 (PMC9973694; doi:10.3201/eid2903.220915)
Supplement: Appendix — Additional information about SARS-CoV-2 infection in a hippopotamus, Vietnam. [file 22-0915-Techapp-s1.pdf]

Article DOI: https://doi.org/10.3201/eid2903.220915

SARS-CoV-2 Infection in a Hippopotamus, Hanoi, Vietnam

Appendix

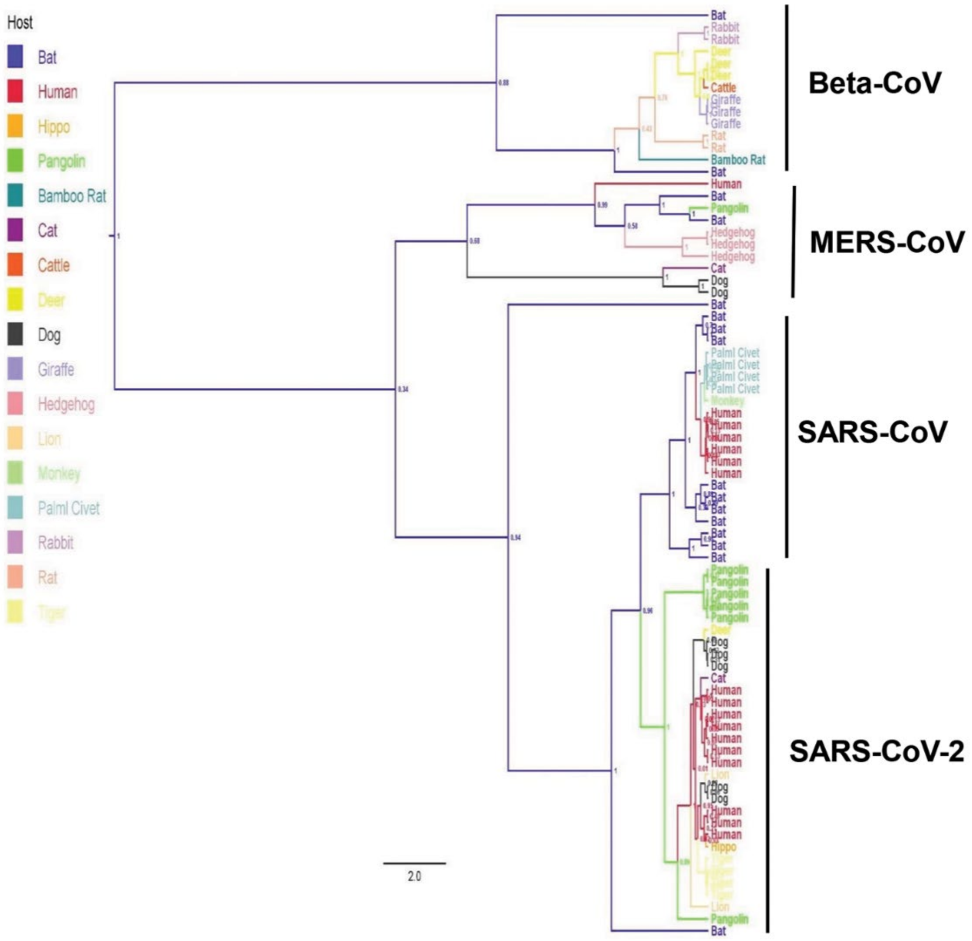

Appendix Figure. The Bayesian maximum clade credibility (MCC) host discrete traits tree for 600 bp RNA-dependent RNA polymerase gene nucleotide sequences of a hippopotamus, 3 human SARS-CoV-2 strains, and other betacoronavirus strains obtained from the GenBank database. The phylogenetic host tree indicated the transmission between betacoronavirus hosts. The number at the node indicates the posterior probability. The bar at the bottom of the figure denotes evolutionary distance. We used the uncorrelated relaxed clock with gamma distribution and the best fit general time reversible plus invariate plus gamma 4 sites (nucleotide substitution model with a constant population size coalescent tree. We ran the Bayesian Markov chain Monte Carlo at 50,000,000 generations and sampled at every 5,000

generations. The effective sample size (ESS) of the analysis was checked by Tracer version 1.6 software (http://tree.bio.ed.ac.uk/software/tracer). The MCC host discrete traits output tree was generated by TreeAnnotator version 1.10.4 (http://tree.bio.ed.ac.uk/software/beast) after burning 10% of the first trees. We reconstructed the host phylogenetic tree using FigTree version 1.4.3 software (http://tree.bio.ed.ac.uk).
